# Elucidating Genetic Drivers of Chronic Inflammation in Obesity

**DOI:** 10.3390/biomedicines14020447

**Published:** 2026-02-17

**Authors:** Leyla O. Rashidova, Danila D. Shashnin, Pavel S. Zubeev, Elena P. Abalikhina, Natalia G. Podprugina, Valeriy A. Kozlov, Sergey V. Stasenko, Tatiana A. Mishchenko, Maria V. Vedunova

**Affiliations:** 1 Institute of Biology and Biomedicine, Lobachevsky State University of Nizhny Novgorod, 23 Gagarin Avenue, 603022 Nizhny Novgorod, Russia; leyla170701@gmail.com (L.O.R.); danila.shashnin@gmail.com (D.D.S.); stasenko@neuro.nnov.ru (S.V.S.); saharnova87@mail.ru (T.A.M.); 2 State Budgetary Healthcare Institution of the Nizhny Novgorod Region City Hospital No. 33, 54 Lenin Avenue, 603076 Nizhny Novgorod, Russia; mlpu33@mail.ru (P.S.Z.); lab33nn@mail.ru (E.P.A.); natalia.gec15@mail.ru (N.G.P.); 3 Prokhorov General Physics Institute of the Russian Academy of Sciences, Vavilov Street 38, 119991 Moscow, Russia; v.kozlov@hotmail.com; 4 Department of Fundamental Sciences, Bauman Moscow State Technical University, 5 2nd Baumanskaya Street, 105005 Moscow, Russia; 5 Moscow Center for Advanced Studies, 20 Kulakova Street, 123592 Moscow, Russia

**Keywords:** obesity, inflammation, cytokine, genetic polymorphism, matrix metalloproteinases, type I collagen, endothelial nitric oxide synthase, angiotensin II receptor type 1 (AGTR1), methylenetetrahydrofolate reductase, cytochrome P450

## Abstract

**Background/Objectives:** Obesity is characterized by chronic low-grade inflammation, which plays a central role in the development of its metabolic complications. The genetic factors influencing this inflammatory phenotype remain incompletely understood. This study aimed to analyze the associations of functional polymorphisms in genes involved in extracellular matrix remodeling (*MMP2*, *MMP9*, *MMP12*, *COL1A1*), metabolism (*MTHFR, CYP3A5*), and vascular regulation (*NOS3*, *AGTR1*) with plasma cytokine profiles and to identify inflammatory subphenotypes in patients with obesity. **Methods:** The study included 127 individuals, comprising 73 patients with excess body weight (body mass index, BMI ≥ 25 kg/m^2^) and 54 individuals with normal weight (BMI 18.5–24.9 kg/m^2^). Genotyping of selected polymorphisms was performed using real-time PCR. Plasma concentrations of 47 cytokines and chemokines were measured by multiplex immunoassay. **Results:** Nominally significant associations between genetic variants and cytokine levels were identified. Polymorphisms *COL1A1* rs1107946 (CA genotype) and *MMP9* rs17576 (AG genotype) were associated with a favorable inflammatory profile (decreased IL-6 and increased IL-10, respectively). In contrast, the *AGTR1* rs5186 (AC genotipe) variant was associated with elevated TNF-α, IP-10/CXCL10, while the *MTHFR* rs1801131 (AC genotipe) variant was linked to increased MIP-1β/CCL4, both reflecting a pro-inflammatory shift. Complex, pleiotropic associations were observed for *MMP2* rs243865 (elevated IL-7 and Fractalkine/CX3CL1) and *NOS3* rs1799983 (elevated MCP-1/CCL2 and Eotaxin/CCL11). Cluster analysis revealed distinct patient subpopulations with differing inflammatory signatures. In one well-defined subgroup, an exploratory model (test R^2^ = 0.537) identified IL-8, IL-15, and albumin as candidate biomarkers predictive of BMI. **Conclusions:** The study identifies candidate genetic polymorphisms and inflammatory biomarkers associated with distinct patterns of systemic inflammation in obesity. These hypothesis-generating findings underscore the phenotypic heterogeneity of obesity and provide a basis for further research into the stratification of patients by the risk of developing metabolic complications.

## 1. Introduction

Obesity is a pathological condition that serves as a risk factor for the development and progression of numerous diseases, including type 2 diabetes mellitus, hypertension, steatohepatitis, reproductive dysfunction, and others [1]. According to the World Health Organization (WHO) estimates, approximately 2.5 billion adults (aged 18 years and older) were overweight in 2022. In 2019, a body mass index (BMI) above the optimal range was responsible for approximately 5 million deaths from non-communicable diseases, including cardiovascular diseases, diabetes, cancer, neurological disorders, chronic respiratory diseases, and digestive system disorders [2].

The role of cytokines is actively studied in the context of the pathogenesis of obesity and its associated complications. Inflammation in obesity contributes to the growth, remodeling, and function of adipose tissue. Accordingly, the negative effects of adipose tissue-associated inflammation are linked to an imbalance between pro-inflammatory and anti-inflammatory systems [3]. In obesity, this balance shifts in favor of pro-inflammatory mediators, resulting in the development of chronic inflammation. Specifically, levels of leptin, resistin, visfatin, tumor necrosis factor-alpha (TNF-α), interleukin-6 (IL-6), and monocyte chemoattractant protein-1 (MCP-1) increase, while levels of adiponectin, adipsin, omentin, IL-10, and IL-4 decrease, promoting a pro-inflammatory state [4]. For example, the pro-inflammatory cytokine TNF-α, whose production increases in obesity, antagonizes the action of adiponectin in lipid metabolism regulation and suppresses its expression [5]. Adiponectin, in contrast, enhances insulin sensitivity and promotes lipid storage in adipose tissue, thereby preventing ectopic fat accumulation [6].

Adipose tissue macrophages are the most prevalent immune cells in adipose tissue. Under normal conditions, adipocytes secrete adiponectin, which promotes polarization towards the alternatively activated M2 macrophages. In obesity, however, adipocytes secrete numerous pro-inflammatory cytokines, including monocyte chemoattractant protein-1 (MCP-1/CCL2), which recruits macrophages and induces their polarization into the pro-inflammatory, classically activated M1 phenotype [7]. M1 macrophages are characterized by the secretion of pro-inflammatory cytokines and enhanced cytotoxic activity. In contrast, M2 macrophages exert anti-inflammatory effects, promote angiogenesis, and attract Th2 T-helper cells and regulatory T cells (Tregs), which serve immunosuppressive functions [8]. In obesity, adipocytes also produce elevated levels of IL-8, a potent neutrophil chemoattractant, contributing to a pro-inflammatory environment [9]. Eosinophils in adipose tissue secrete IL-4, IL-5, IL-6, and IL-13, which possess anti-inflammatory properties and help maintain immune balance and M2 macrophage polarization. However, obesity is associated with a decrease in eosinophil numbers and a concomitant increase in M1 macrophages in adipose tissue [10]. Adipose tissue also contains regulatory B cells that secrete the anti-inflammatory cytokine IL-10. In obesity, there is a reduction in these cells and an increase in mature B cells, which promote inflammation through the production of immunoglobulin G, IL-6, IL-8, and TNF-α [11].

One potential trigger of inflammation in obesity is an increased influx of nutrients. Free fatty acids and lipoproteins can stimulate toll-like receptor 4-mediated activation of caspase-1, which participates in the conversion of IL-1β from its precursor form. Elevated glucose levels promote oxidative stress, which activates cryopyrin and subsequently triggers caspase-1 activation [12].

Adipocyte hypertrophy and impaired adipose tissue vascularization, commonly observed in obesity, contribute to the development of microischemic areas. In non-ischemic regions, increased oxygen consumption occurs due to the activation of adenine nucleotide translocase 2 (ANT-2), a translocase located on mitochondrial membranes, by free fatty acids, resulting in the uncoupling of oxidative metabolism. This induces relative hypoxia in adipocytes, which, through the activation of hypoxia-inducible factor-1-alpha (HIF-1α), stimulates the synthesis of chemokines such as MCP-1, TNF-α, and IL-6. These chemokines, in turn, promote macrophage accumulation in adipose tissue [13].

Although there is evidence for the heritability of body mass index and a genetic contribution to individual predisposition to weight gain, currently identified single-nucleotide polymorphisms (SNPs) account for less than 3% of the variance in obesity susceptibility [14]. Given that classifications based solely on anthropometric indicators do not adequately capture the risk of developing obesity-related complications, phenotyping based on genetic and molecular markers presents a promising approach for prognostication and the development of personalized treatment strategies [15].

This study focuses on the analysis of functionally significant gene polymorphisms involved in key pathogenic mechanisms of obesity-related complications and the formation of the inflammatory profile characteristic of obesity.

The matrix metalloproteinase 9 (*MMP9*) gene polymorphism rs17576 (Arg279Gln), located in the coding region, influences enzymatic activity. The GG genotype is associated with reduced MMP9 levels in obese children [16], while the A allele correlates with increased expression in adipose tissue [17]. The *MMP12* rs652438 (Asn357Ser) variant, located in the hemopexin domain, alters enzymatic activity, with the G allele linked to reduced protease activity [18] and an increased risk of abdominal obesity accompanied by systemic inflammation [19]. The *MMP2* rs243865 (C-1306T) SNP, located in the promoter region at the Sp1 binding site, reduces transcriptional activity in the presence of the T allele [20], thereby influencing the enzyme’s anti-inflammatory functions [21]. The *COL1A1* rs1107946 variant in the promoter region of the collagen type I alpha-1 chain gene may regulate the expression of type I collagen, which in turn influences adipogenesis via YAP signaling and modulates adiponectin expression [22,23].

Genes involved in the regulation of vascular function also play a critical role. The nitric oxide synthase (*NOS3*) gene variant rs1799983 (Glu298Asp), encoding endothelial nitric oxide synthase (eNOS), leads to reduced nitric oxide (NO) bioavailability [24], with the T allele associated with elevated levels of pro-inflammatory cytokines [25]. The angiotensin II type I receptor (AGTR1) gene polymorphism rs5186 (A1166C), located in the 3′-untranslated region, results in increased receptor expression [26] and is associated with insulin resistance and a pro-inflammatory state [27].

Metabolic and biochemical processes are intimately linked with the pathogenesis of complications arising from excessive body weight. The methylenetetrahydrofolate reductase (*MTHFR*) gene polymorphism rs1801131 (A1298C) in exon 7 reduces enzymatic activity [28], contributing to hyperhomocysteinemia and an increased risk of metabolic disorders [29]. The cytochrome P450 family 3 subfamily A member 5 (*CYP3A5*) gene variant rs776746 (A > G in intron 3) introduces an alternative splicing site that leads to the formation of a premature stop codon [30], resulting in decreased gene expression in the context of obesity and inflammation [31,32].

Therefore, the aim of this study was to analyze the associations of functionally significant polymorphisms in genes involved in extracellular matrix remodelling (*MMP2*, *MMP9*, *MMP12*, *COL1A1*), metabolism (*MTHFR*, *CYP3A5*), and vascular regulation (*NOS3*, *AGTR1*) with plasma cytokine profiles in patients with obesity.

## 2. Materials and Methods

### 2.1. Materials

The study group comprised 127 individuals aged 20 to 79 years (mean age 45.82 ± 15.33), including 73 individuals with excess body weight (BMI ≥ 25 kg/m^2^, mean BMI 36.49 ± 9.38) and 54 individuals with a normal BMI (18.5–24.9 kg/m^2^, mean BMI 22.35 ± 2.49) according to WHO criteria. Of the participants, 76% were women and 24% were men. The exclusion criteria comprised acute-stage chronic somatic or oncological diseases and acute respiratory viral infections at the time of examination. Data on chronic comorbidities (e.g., hypertension, type 2 diabetes), smoking status, and alcohol consumption were collected via structured interview with cross-reference to available medical records (Table S2). Specific data on concomitant medication use were not systematically collected.

The study was conducted in accordance with the ethical principles of medical research involving human subjects as outlined in the 9th revision of the Declaration of Helsinki (World Medical Association, October 2013). The study protocol was approved by the Local Ethics Committee (Protocol No. 1, dated 2 December 2020). All participants provided written informed consent.

Venous blood sampling for investigation was performed in the morning on an empty stomach after an 8–12 h overnight fast. Blood samples were collected from study participants using VACUETTE vacuum tubes containing K3-EDTA and lithium heparin (Greiner Bio-One, Kremsmünster, Austria). Plasma was obtained by centrifuging the blood samples at 3000 rpm for 10 min. The samples were subsequently stored at −80 °C.

### 2.2. Detection of Polymorphisms

Genomic DNA was extracted from whole blood cells using the DNA-Extran-1 reagent kit in accordance with the manufacturer’s instructions (Syntol, Moscow, Russia). Specificity and reproducibility of genotyping were controlled by including positive controls with verified genotypes and negative controls (i.e., reaction mixture without template DNA) in each analysis. Genotyping quality was validated by repeat analysis of 10% randomly selected samples.

The following polymorphisms were analyzed using commercially available kits: *MMP2* (rs243865)*, MMP9* (rs17576)*, MMP12* (rs652438)*, NOS3* (rs1799983)*, MTHFR* (rs1801131)*, CYP3A5* (rs776746), *COL1A1* (rs1107946), and *AGTR1* (rs5186) (Syntol, Russia).

### 2.3. Multiplex Analysis

Plasma levels of sCD40L, EGF, eotaxin, FGF-2, FLT-3L, fractalkine, G-CSF, GM-CSF, GROa, IFNa2, IFNy, IL-1a, IL-1b, IL-1RA, IL-2, IL-3, IL-4, IL-5, IL-6, IL-7, IL-8, IL-9, IL-10, IL-12 (p40), IL-12 (p70), IL-13, IL-15, IL-17A, IL-17E|IL-25, IL-17F, IL-18, IL-22, IL-27, IP-10, MCP-1, MCP-3, M-CSF, MDC, MIG, MIP-1a, MIP-1β, PDGF-AA, PDGF-AB|BB, TGFa, TNFa, TNFb, and VEGF-A were quantified via multiplex analysis using a MAGPIX immunoassay system (Luminex, Austin, TX, USA) and the MILLIPLEX 48-plex human cytokine/chemokine panel system (Merck, Rahway, NJ, USA). The limits of detection (LOD) for each cytokine, defined as the minimum detectable concentration plus two standard deviations (MinDC+2SD) as provided in the manufacturer’s specification, are presented in Table S1. The LOD values ranged from 0.20 pg/mL (IL-6) to 52.07 pg/mL (IL-17E/IL-25) for the overnight protocol. Cytokine concentrations were calculated from median fluorescence intensity (MFI) data using the Luminex xPONENT^®^ software (version 4.2). In this system, samples with MFI below the detection threshold of the standard curve are automatically reported by the software as a non-numerical value below the limit of detection (e.g., ‘<10 pg/mL’). Values below the limit of detection (LOD) were imputed as LOD/√2.

### 2.4. Biochemical Analysis

Biochemical markers (albumin, creatinine, glucose, C-reactive protein, and alkaline phosphatase) were determined using a DIRUI CS-T240 automated analyzer (Dirui Industrial Co., Ltd., Changchun, China) and DiaSys reagent kits (Diagnostic Systems GmbH, Holzheim, Germany). Sample preparation and analysis were conducted in accordance with the manufacturers’ protocols.

### 2.5. Statistical Data Processing

All statistical analyses were conducted in the Google Colab cloud environment using Python version 3.11. The following specialized packages were employed: SciPy (version 1.13.0) for basic statistical testing, statsmodels (version 0.15.0) for advanced modeling, and Pandas (version 2.2.0) for data processing. Visualizations were generated using Matplotlib (version 3.8.0) and Seaborn (version 0.13.0).

Differences in allele and genotype frequency distributions between groups, as well as conformity to the Hardy–Weinberg equilibrium in the control group, were assessed using the χ^2^ test.

Associations between gene polymorphisms and cytokine levels were assessed using a comprehensive regression-based approach. A threshold of *n* ≥ 10 per genotype group was applied for all association tests. The primary analysis was performed using ordinary least squares (OLS) linear regression on log-transformed data (natural logarithm), adjusted for age, sex, body mass index (BMI), glucose, triglycerides, and high-density lipoprotein cholesterol. To ensure the robustness of results and test sensitivity to different distributional assumptions, the following additional methods were applied: robust Huber regression (outlier-resistant), rank-based analysis (nonparametric rank regression), and Box–Cox transformed data analysis. A sensitivity analysis was conducted with additional adjustment for lifestyle factors (smoking, alcohol consumption, presence of chronic diseases). Values below the limit of detection were handled using the standard substitution approach, LOD/√2. A reliable association was defined by the simultaneous fulfillment of the following criteria: statistical significance (*p* < 0.05) in the primary OLS model, retained significance (*p* < 0.05) in alternative models (robust, rank-based, Box–Cox). For visualization, boxplots displaying raw cytokine concentrations (pg/mL) were used. Results are presented as effect sizes: β coefficients with 95% confidence intervals. For each significant association, the best-fitting genetic model (additive, dominant, or recessive) was determined using the Akaike Information Criterion (AIC), and results are reported accordingly (Table S3).

The false discovery rate (FDR) was controlled across all tests using the Benjamini–Hochberg procedure at FDR < 0.05.

To assess gene–gene interaction according to three a priori biologically grounded hypotheses, we used generalized linear models (Gamma distribution, logarithmic link function) adjusted for age, sex, and BMI. The analysis examined interactions for *NOS3 × AGTR1* (IL-6, TNF-α, MCP-1, IP-10), *MMP9 × Col1A1* (IL-6, TNF-α, TGF-α, VEGF-A, IL-1β), and *MTHFR×CYP3A5* (IL-6, TNF-α, IL-1β, IL-8, IL-18). To ensure robustness of the analysis, the minimum group size for a combined genotype was set at n ≥ 3. Statistical significance was assessed at a two-tailed *p* < 0.05.

Prior to analyzing the associations between gene polymorphisms and cytokine levels, the potential confounding effects of demographic and anthropometric variables (age, sex, and BMI) were evaluated. Correlations between continuous variables (BMI, age) and cytokine levels were assessed using Spearman’s rank correlation coefficient for nonparametric data. Correlation analysis between biochemical parameters and BMI or age was performed using Spearman’s correlation coefficient and Pearson’s correlation coefficient.

In all analyses, statistical significance for nominal associations was set at a two-sided *p*-value < 0.05.

To develop a model linking BMI and inflammatory status, a comprehensive data analysis was performed, commencing with the preprocessing of an initial dataset comprising 71 observations (from the total cohort of 127) and 67 features. Only subjects with a complete set of candidate feature measurements were included to ensure model stability. Since 27 features contained over 20% missing values, a preliminary selection of 39 features with no more than 20% missing data was conducted. Visualization revealed the presence of outliers (Figure S4), which were addressed using the robust Winsorization method based on the interquartile range (IQR). The boundaries were defined as Q1—1.5 × IQR and Q3 + 1.5 × IQR, and extreme values were replaced with these threshold values, thereby minimizing the influence of outliers without data loss. Missing values were imputed using a hybrid strategy: for features with ≤10% missing data, median imputation was used, while for the 10–20% range, the k-nearest neighbors (KNN) method was applied. The categorical variable ‘sex’ was binarized (0 for male, 1 for female). Following preprocessing, an analysis of the correlation matrix (Figure S5) revealed significant multicollinearity, which was mitigated through sequential feature selection using a Variance Inflation Factor (VIF) threshold of 5 (Figure S6). Initial training of ensemble regressors (Random Forest, KNN, Bagging, Gradient Boosting, XGBoost) on the entire dataset yielded unsatisfactory performance (high MAE, negative R^2^), indicating data heterogeneity and leading to the clustering hypothesis. Hierarchical clustering confirmed the presence of at least two distinct clusters. To prevent data leakage and assess generalization, the data for Cluster 1 were split into training (70%) and test (30%) sets prior to any feature engineering or model training. All subsequent steps (preprocessing, feature selection, clustering, model training) were confined to the training set. Subsequently, Ridge regression (to assess linear relationships) and CatBoost (to capture non-linearities) were trained separately for each cluster, with an additional stacked model constructed for Cluster 1. Final model performance was evaluated on the held-out test set (Table S6, Figure S7). The interpretation of feature importance via SHAP analysis ultimately identified distinct inflammatory patterns associated with BMI.

## 3. Results

Analysis of the influence of demographic and anthropometric factors revealed limited statistically significant correlations between cytokine levels and BMI after false discovery rate (FDR) correction for multiple comparisons. Only two cytokines, IL-17E/IL-25 (r = 0.44, FDR-adjusted *p* = 0.023) and IL-27 (r = −0.40, FDR-adjusted *p* = 0.023) (Figure S1), demonstrated significant associations with BMI (Table S4). No significant correlations with age were observed. At the initial stage, we also assessed the association of the studied polymorphic variants with key clinical and laboratory parameters associated with obesity. No statistically significant associations were identified between genotypes and BMI or key biochemical parameters. Correlation analysis confirmed the expected associations: glucose and C-reactive protein concentrations were positively correlated with age but not with BMI, whereas creatinine and alkaline phosphatase levels were dependent on both variables (Figure S2). Furthermore, a complementary Pearson correlation analysis yielded no significant associations (Figure S3), suggesting that the identified relationships are monotonic but non-linear in nature. Given the replication of these expected correlations, we proceeded to analyze more specific predictor–cytokine profiles.

For polymorphisms with a low frequency of the minor allele (such as *MMP12* (rs652438) and *CYP3A5* (rs776746)), no homozygous carriers of the minor allele were detected in the study groups, which is statistically expected for rare alleles in limited sample sizes. All investigated polymorphisms were in Hardy–Weinberg equilibrium in the control group (Table S5) and showed no significant differences in genotype distribution between the control and obese groups (all *p* > 0.05, χ^2^ test).

No significant associations emerged for *MMP12* rs652438 (lacking minor allele homozygotes) or *CYP3A5* rs776746 (limited by small AG subgroup, n = 8), highlighting sample size constraints for rare variants. It is important to note that none of the associations reported in this section survived a stringent global false discovery rate (FDR) correction for the multiple comparisons across all tested SNP-cytokine pairs. Therefore, the associations reported in this section are presented as nominally significant (*p* < 0.05) and should be considered hypothesis-generating, pending validation in larger, independent cohorts. Given the sample size, a post hoc analysis of statistical power was conducted. The study had an estimated 80% power (α = 0.05) to detect only large genetic effects (Cohen’s *d* ≥ 0.66) in the obesity cohort. Analysis of the nominally significant associations revealed that most were underpowered (post hoc power < 50%). The exception was the association of *AGTR1* rs5186 with IP-10 levels, which exhibited a large effect size (Cohen’s *d* = 1.55) and adequate power. This underscores that the study was primarily sensitive to large genetic effects and reinforces the preliminary, hypothesis-generating nature of the findings presented below.

**Figure 1:**
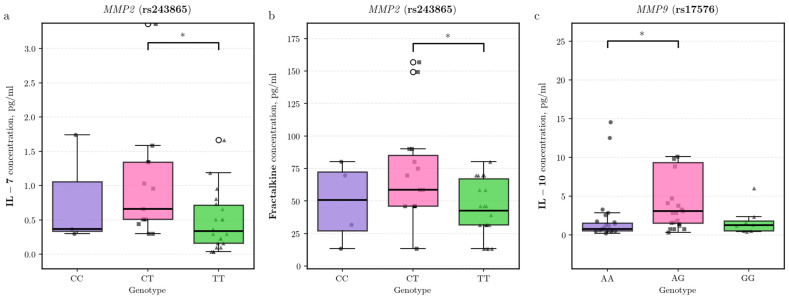
Association of matrix metalloproteinase gene polymorphisms with cytokine levels in obesity cohort. Plasma levels of (**a**) IL-7 and (**b**) Fractalkine (CX3CL1) across *MMP2* (rs243865) genotypes, and (**c**) IL-10 across *MMP9* (rs17576) genotypes. Statistical significance was assessed using covariate-adjusted linear regression on log-transformed data (* *p* < 0.05).

We identified potential associations between polymorphisms in extracellular matrix-related genes and cytokine concentrations. For the *MMP2* rs243865 polymorphism, CT heterozygotes exhibited elevated IL-7 levels compared to TT homozygotes (β = 0.86, 95% CI [0.17, 1.56], *p* = 0.016; n: CT = 16, TT = 24) (Figure 1a). A similar pattern was observed for Fractalkine (CX3CL1), where CT heterozygotes showed higher levels relative to TT homozygotes (β = 0.56, 95% CI [0.10, 1.02], *p* = 0.018) (Figure 1b).

For the *MMP9* rs17576 polymorphism, analysis indicated that AG heterozygotes had higher plasma levels of the anti-inflammatory cytokine IL-10 compared to AA homozygotes (β = −0.85 for AA vs. AG, 95% CI [−1.60, −0.09], *p* = 0.028; n: AG = 32, AA = 31) (Figure 1c).

**Figure 2:**
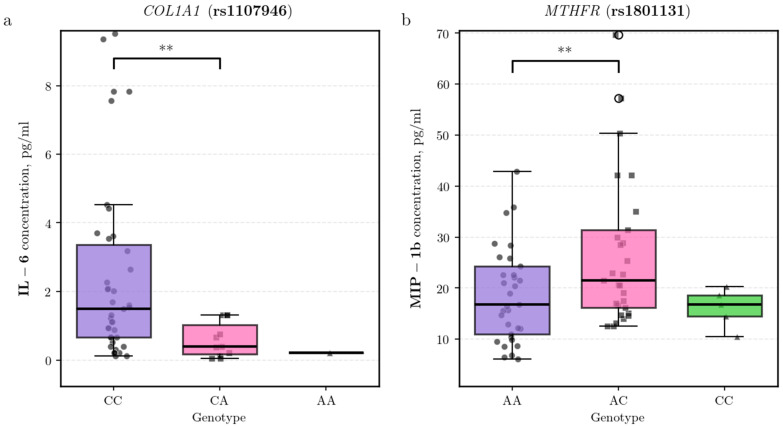
Association of single-nucleotide polymorphisms with cytokine levels in obesity cohort. Plasma levels of (**a**) IL-6 across *COL1A1* (rs1107946) genotypes and (**b**) MIP-1β (CCL4) across MTHFR (rs1801131) genotypes. Statistical significance was assessed using covariate-adjusted linear regression on log-transformed data (** *p* < 0.01).

Continuing the analysis of other genetic pathways, we identified nominally significant associations for structural and metabolic genes. For the *COL1A1* rs1107946 polymorphism, CA heterozygotes had significantly lower plasma levels of pro-inflammatory IL-6 compared to CC homozygotes (β = −1.33, 95% CI [−2.22, −0.45], *p* = 0.004; n: CA = 18, CC = 54) (Figure 2a). For the *MTHFR* rs1801131 polymorphism, AC heterozygotes exhibited elevated concentrations of the chemokine macrophage inflammatory protein-1 beta (MIP-1β/CCL4) relative to AA homozygotes (β = 0.36, 95% CI [0.11, 0.61], *p* = 0.005; n: AC = 34, AA = 35) (Figure 2b).

**Figure 3:**
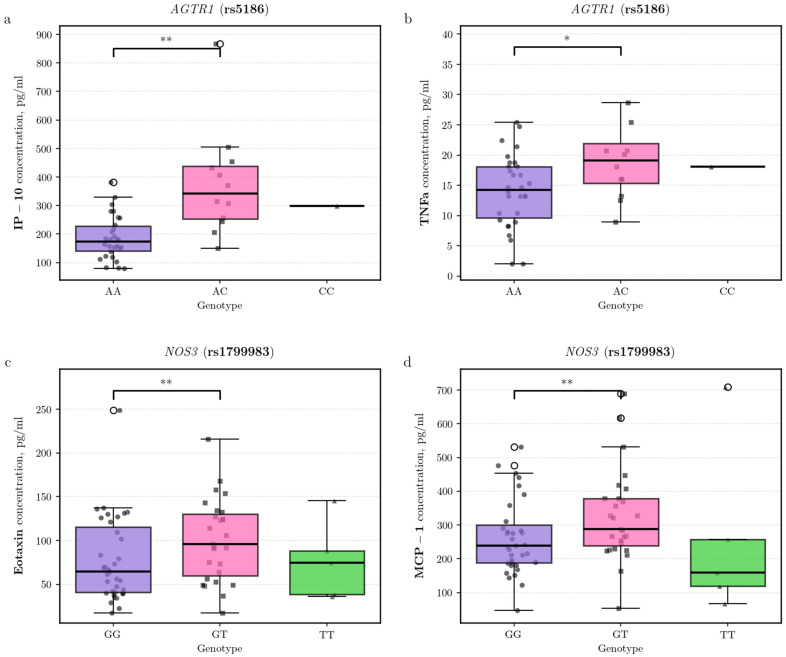
Association of vascular regulation gene polymorphisms with plasma cytokine levels in obesity cohort. Plasma concentrations of (**a**) IP-10 (CXCL10) and (**b**) TNF-α across *AGTR1* (rs5186) genotypes, and (**c**) eotaxin (CCL11) and (**d**) MCP-1 (CCL2) across *NOS3* (rs1799983) genotypes. Statistical significance was assessed using covariate-adjusted linear regression on log-transformed data (* *p* < 0.05, ** *p* < 0.01).

Given that complications of obesity, such as hypertension, are closely linked to vascular function, the analysis of genes involved in vascular regulation was of particular interest. For the *AGTR1* rs5186 polymorphism, AC heterozygotes showed significantly higher plasma levels the pro-inflammatory cytokine TNF-α and the chemokine interferon gamma-induced protein 10 (IP-10/CXCL10) compared to AA homozygotes (IP-10: β = 0.99, 95% CI [0.26, 1.72], *p* = 0.009; n: AC = 12, AA = 32; TNF-α: β = 0.56, 95% CI [0.03, 1.08], *p* = 0.038) (Figure 3a,b). Similarly, for the *NOS3* (eNOS) rs1799983 polymorphism, GT heterozygotes exhibited elevated concentrations of eotaxin (CCL11) and MCP-1 (CCL2) relative to GG homozygotes (eotaxin: β = 0.79, 95% CI [0.23, 1.35], *p* = 0.006; n: GT = 27, GG = 40; MCP-1: β = 0.55, 95% CI [0.16, 0.94], *p* = 0.007; n: GT = 27, GG = 40) (Figure 3c,d).

**Figure 4:**
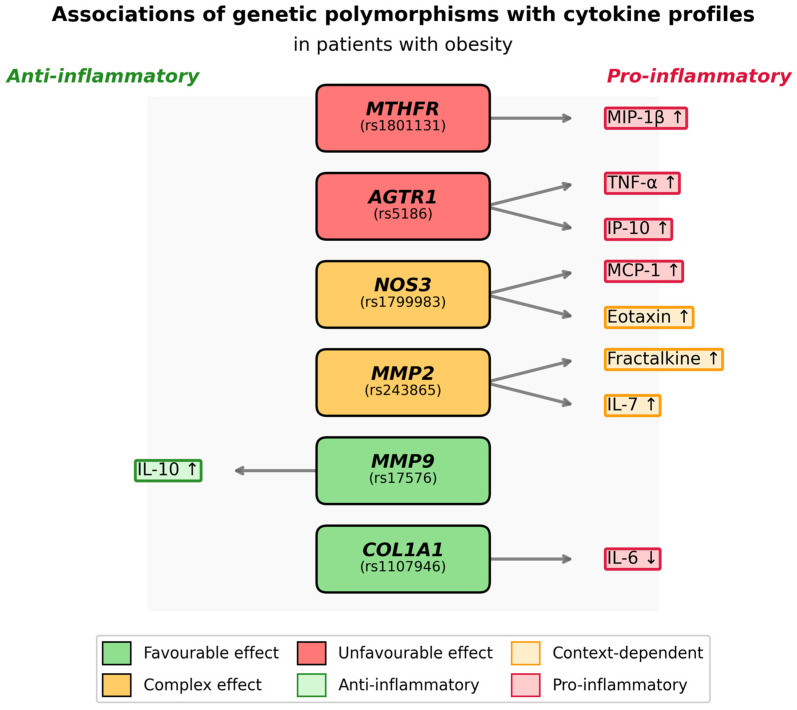
Integrative schematic representation of SNP associations with cytokine profiles in patients with obesity.

In summary, the identified associations reveal consistent patterns in the influence of genetic variants on inflammatory status in obesity. To provide a comprehensive visualization of all significant associations, a summary diagram was developed, illustrating the direction of the effects of the studied polymorphisms on the cytokine profile (Figure 4).

Analysis of three a priori biologically grounded gene–gene interactions did not reveal statistically significant effects on cytokine levels. The absence of significant interactions, despite their biological plausibility, may be attributed to the limited sample size for certain genotype combinations.

Given the limited associations found in the initial correlation analysis, we hypothesized that the heterogeneity of the patient population might be obscuring stronger, subgroup-specific relationships between inflammatory markers and BMI. To test this, we employed a cluster-then-predict framework to identify latent subgroups within the data before constructing regression models.

**Figure 5:**
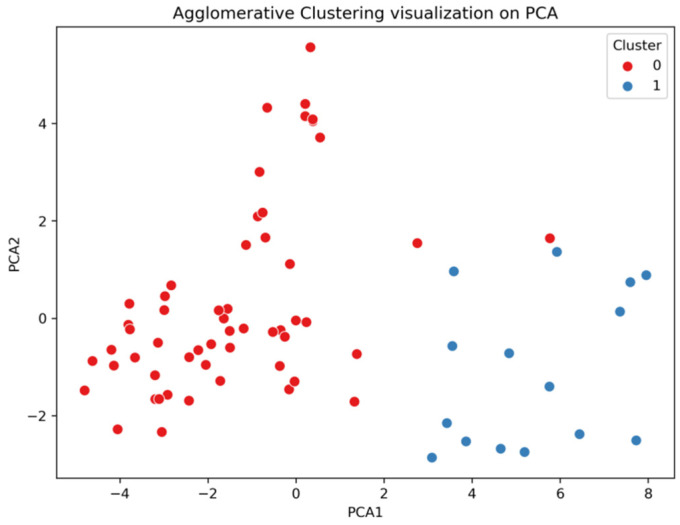
Hierarchical clustering of the training data projected onto the first two principal components reveals two distinct subgroups: Cluster 0 (subgroup with non-linear/complex BMI–inflammation relationships) and Cluster 1 (subgroup with more linear BMI–inflammation relationships).

**Figure 6:**
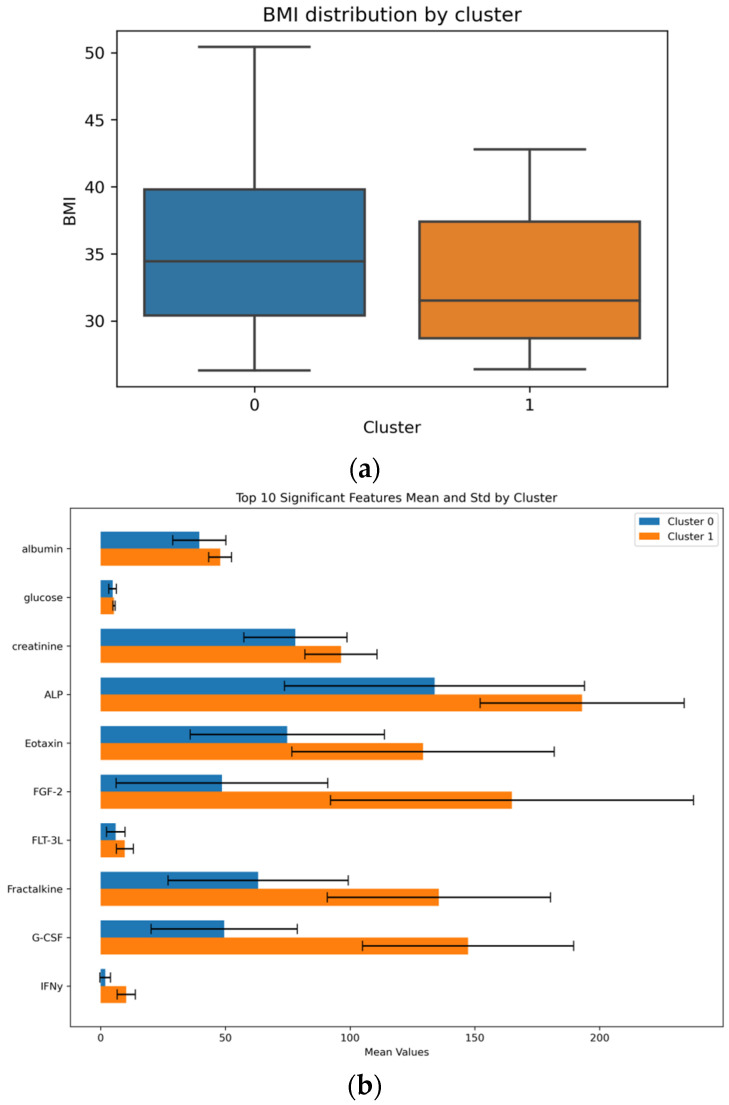
Comparative analysis of the identified clusters. (**a**) BMI distribution in Cluster 0 (non-linear/complex subgroup) and Cluster 1 (linear subgroup). (**b**) The 10 most statistically distinct features (independent two-sample *t*-test) between the two subgroups.

For the identification of clusters within the data, the initial training of ensemble regressors on the entire training dataset showed unsatisfactory results with high MAE and negative R^2^, which indicated the necessity of accounting for the internal data structure and led to the application of hierarchical clustering. This analysis revealed at least two stable clusters with visually confirmed separability in the space of principal components (Figure 5); a comparative analysis of the clusters (Figure 6) demonstrated their statistically significant differences both in the distribution of BMI (Figure 6a) and in the values of the 10 most significant features according to the *t*-test (Figure 6b).

**Figure 7:**
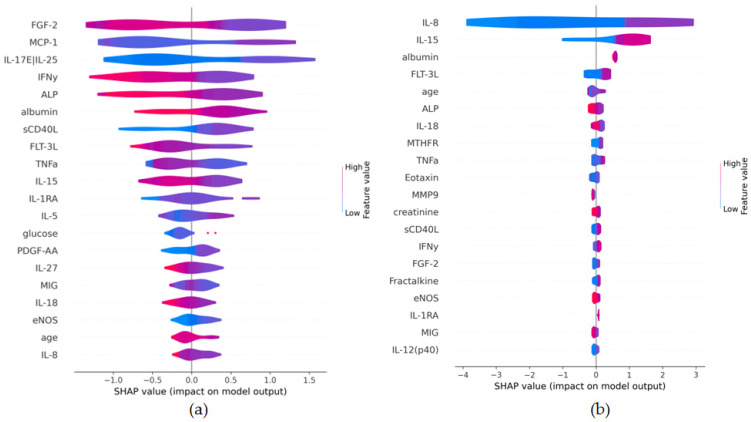
Feature importance in (**a**) cluster 0 (non-linear/complex subgroup) and (**b**) cluster 1 (linear subgroup).

The results of predictive modelling differed substantially between clusters: in Cluster 0, Ridge regression was inapplicable (R^2^ < 0), and CatBoost showed modest results (MAE: 4.973, R^2^: 0.149), whereas in Cluster 1, all models demonstrated a reproducible predictive signal on an independent test set—CatBoost (MAE: 3.062, R^2^: 0.249), Ridge (MAE: 2.580, R^2^: 0.528) and the combined stacking model (MAE: 2.179, R^2^: 0.537) (Table S6). Analysis of feature importance via SHAP (Figure 7) revealed in Cluster 0 (Figure 7a) a distributed influence of multiple features without clear dominance, while in Cluster 1 (Figure 7b), the three most influential features were clearly identified: IL-8, IL-15, and albumin.

## 4. Discussion

Our analysis revealed a general lack of strong correlations between cytokine levels and key demographic parameters (BMI and age), as well as the absence of associations between the studied polymorphic variants and clinical or laboratory parameters related to obesity. At the same time, correlation analysis reproduced the expected associations of conventional markers (glucose, creatinine, and alkaline phosphatase) with age and BMI, thereby validating the quality of the collected data and the appropriateness of the statistical approach. These findings support the interpretation of subsequent genetic associations with cytokine profiles as independent of key anthropometric and clinical variables, reinforcing the significance of the observed relationships and highlighting the direct role of genetic variants in regulating inflammatory status.

Obesity is not merely characterized by an increase in adipocyte number, but also by extensive remodeling of adipose tissue. Inflammatory processes in adipose tissue stimulate the production of MMPs by adipocytes and immune cells. Conversely, an imbalance in MMP activity contributes to adipose tissue fibrosis, which impairs adipocyte function and exacerbates inflammation [33,34]. Accordingly, we focused on investigating the associations between cytokine levels and single-nucleotide polymorphisms in matrix metalloproteinase genes: *MMP2* (rs243865), *MMP9* (rs17576), and *MMP12* (rs652438).

Analysis of the *MMP9* (rs17576) polymorphism revealed that carriers of the heterozygous AG genotype had elevated levels of the anti-inflammatory cytokine IL-10 compared to AA homozygotes. This finding suggests that this genetic variant may contribute to modulating inflammatory pathways in obesity. This specific immunophenotype (elevated IL-10) aligns with the more favorable systemic profile—including lower biological age acceleration and improved vascular health parameters—previously reported for the AG genotype in an independent study [35].

Analysis of the *MMP2* (rs243865) polymorphism revealed that the heterozygous CT genotype is associated with elevated levels of IL-7 and fractalkine (CX3CL1) compared to the homozygous TT genotype. This observation is consistent with the functional role of the T allele, which reduces the transcriptional activity of the gene promoter [36], and suggests a dose-dependent influence of the variant on *MMP2* expression. Interpreting these associations requires consideration of the biological context of the cytokines. The elevated level of Fractalkine, a key adipochemokine associated with insulin resistance in obesity [37], receives direct explanation, as MMP2 is the primary metalloproteinase responsible for the proteolytic release (shedding) of its active form under inflammatory conditions [38]. For IL-7, which, according to mouse models, may exert protective metabolic effects [39], a direct regulatory mechanism via MMP2 has not been established; however, other members of the MMP family (e.g., MMP9) are capable of cleaving this cytokine [40]. Thus, our results demonstrate that the heterozygous CT genotype of the *MMP2* (rs243865) polymorphism is associated with elevated levels of two cytokines playing different roles in inflammation and metabolism. This association is consistent with functional data on the polymorphism, as well as with literature data describing the involvement of MMP2 in fractalkine processing and the potential involvement of metalloproteinases in IL-7 regulation. Our findings contribute to understanding how natural genetic variations in protease genes may be related to the variability of immune and cytokine status in obesity.

Shifting from enzymatic components to structural elements of the extracellular matrix, the association identified for the type I collagen gene (*COL1A1*) is noteworthy. The rs1107946 polymorphism in the *COL1A1* gene was associated with the heterozygous CA variant, which showed decreased IL-6 levels compared to the CC genotype. Type I collagen suppresses adipogenesis by downregulating the expression of C/EBPα and PPARγ and can influence immune cell infiltration into adipose tissue [41]. In obesity, *COL1A1* expression is increased, contributing to fibrosis and amplifying inflammatory responses [42]. IL-6 functions as a proinflammatory cytokine that promotes insulin resistance by activating the Janus kinase-signal transducer and activator of transcription (JAK-STAT) signaling pathway, upregulating suppressor of cytokine signaling 3 (SOCS3), and inhibiting phosphoinositide 3-kinase (PI3K) signaling [43,44]. Therefore, the lower IL-6 levels associated with the CA genotype may reflect a modulation of collagen-mediated pathways that results in a relatively attenuated inflammatory response. This suggests a potential protective effect of the heterozygous CA substitution in mitigating obesity-associated inflammation.

The *MTHFR* (methylenetetrahydrofolate reductase) gene is a candidate for modulating inflammatory processes in obesity, with its influence extending to the metabolic pathways underlying the condition itself. Impaired *MTHFR* function can affect folate metabolism, and experimental evidence links this dysregulation to increased adiposity on a high-fat diet [45] and to potential changes in eating behavior through altered neurotransmitter synthesis [46,47]. Furthermore, the rs1801131 polymorphism is associated with decreased enzyme activity [28], which can elevate homocysteine—a metabolite generally linked to a proinflammatory state [48,49]. Within this framework, our study identifies a specific immunomodulatory association: the heterozygous AC genotype was linked to elevated plasma levels of the chemokine MIP-1β (CCL4). As MIP-1β is a critical driver in recruiting immune cells to adipose tissue and promoting obesity-induced inflammation [50], our finding suggests that this *MTHFR* variant may be associated with a more pronounced chemokine-mediated inflammatory response.

It is important to note that in humans, obesity and insulin resistance are characterized by endothelial dysfunction, which may be associated with endothelial nitric oxide synthase deficiency [51]. Endothelial nitric oxide synthase (eNOS), encoded by the *NOS3* gene, plays a role in inflammation. For example, the enzyme can regulate the expression of proinflammatory molecules, including nuclear factor-κB (NF-κB) and cyclooxygenase-2 [52]. It has previously been reported that the T allele of the rs1799983 polymorphism shows an increased association with inflammation, manifested by higher levels of interleukin 1, interleukin 6, and tumor necrosis factor alpha [25]. Our findings are consistent with this view, as we observed that the heterozygous GT genotype was associated with increased plasma concentrations of the chemokines eotaxin (CCL11) and MCP-1 (CCL2). Eotaxin, a classical mediator of allergic inflammation and eosinophil recruitment, may also contribute to the maintenance of metabolic homeostasis by modulating inflammation in adipose tissue [53]. MCP-1, in turn, is a potent driver of monocyte and macrophage infiltration and is strongly associated with obesity and insulin resistance. Interestingly, experimental data from a murine model indicate that eNOS plays a protective role by limiting MCP-1 production in the context of inflammatory kidney disease [54]. Thus, our observed association of the *NOS3* GT genotype with elevated MCP-1 levels may reflect a correlation between this genetic variant and a more pronounced proinflammatory chemokine profile characteristic of obesity. Further functional studies are required to establish a causal relationship and elucidate the underlying mechanisms.

The angiotensin II type 1 receptor (*AGTR1*), expressed by adipocytes and immune cells, is a key mediator of the renin-angiotensin system (RAS) [55]. Its rs5186 polymorphism has been associated with conditions of RAS hyperactivation, such as hypertension and metabolic syndrome [56]. In our cohort, the heterozygous AC genotype of this polymorphism was associated with elevated plasma levels of the chemokine IP-10 (CXCL10) and the cytokine TNF-α. This finding is mechanistically supported by experimental evidence demonstrating that angiotensin II signaling through the *AGTR1* receptor directly induces IP-10 expression in endothelial cells [57]. IP-10 is a potent chemoattractant for T cells and a key player in vascular inflammation, while TNF-α is a master regulator of systemic inflammation and insulin resistance. Thus, our results provide evidence linking the *AGTR1* rs5186 AC genotype to an enhanced pro-inflammatory state. This suggests a potential immunological mechanism—characterized by elevated levels of IP-10 and TNF-α—through which this genetic variant may contribute to the development of metabolic complications in obesity.

To elucidate the relationship between inflammatory status and BMI amid substantial data heterogeneity, we implemented a clustering-based modelling strategy. This approach revealed distinct patient subpopulations, which was critical for building exploratory predictive models. The obtained results demonstrate the fundamental importance of accounting for this data heterogeneity. The inability to construct an accurate model on the entire dataset and the subsequent identification of clusters indicate the presence of distinct pathophysiological patterns or subtypes within the studied population.

The difference in model efficacy between the clusters is a key finding of our study. The relatively higher performance metrics of Ridge regression in Cluster 1 indicate that the relationship between inflammatory markers and BMI in this patient group has a pronounced linear component. At the same time, the data in Cluster 0 demonstrate a complex, non-linear nature, which could not be adequately described with high accuracy by either the linear model or the tree-based ensemble.

Furthermore, the analysis of feature importance using SHAP provides a biologically interpretable result. In Cluster 1, the model clearly identifies the leading role of specific cytokines (IL-8, IL-15) and albumin in predicting BMI. This allows for the formulation of hypotheses about the potential mechanisms linking inflammation and metabolic processes, specifically in this subpopulation. In contrast, the diffuse contribution of numerous features in Cluster 0 may indicate either more complex and cross-cutting interactions or that key predictors were not accounted for in the original dataset. While a substantial gap between training and test performance is evident (Table S6, Figure S8), the models’ ability to outperform a permutation null on the test set confirms a reproducible signal, supporting the exploratory findings while underscoring the need for validation in larger cohorts.

Our results contribute to the growing evidence of genetic modulation of inflammation in obesity and underscore the phenotypic heterogeneity of this condition. From a research perspective, these insights lay the groundwork for developing stratified approaches that move beyond a one-size-fits-all view of obesity-associated inflammation. The nominally significant associations between polymorphisms in extracellular matrix, vascular, and metabolic pathways with distinct cytokine profiles, alongside the identification of patient subpopulations via cluster analysis, suggest a potential framework for refining inflammatory phenotyping in obesity beyond anthropometric measures. While our findings are preliminary and require validation, they point to specific hypotheses. For example, future studies could test whether individuals with combined genetic and inflammatory profiles associated with a pro-inflammatory state (e.g., *AGTR1* rs5186 AC genotype with elevated TNF-α/IP-10) represent a distinct inflammatory endotype. To advance this line of inquiry, our findings highlight the need for further research to replicate these associations in larger cohorts, to explore the underlying molecular mechanisms, and to determine the longitudinal stability and broader metabolic correlates of these inflammatory signatures. This study thus provides a set of candidate markers and hypotheses that can guide subsequent efforts to develop a more refined, mechanism-based understanding of obesity.

### Study Limitations

This study has several limitations that should be considered when interpreting the results. The relatively small sample size limits the statistical power to detect small-to-moderate effect sizes, especially for polymorphisms with low minor allele frequency. As detailed in Section 3, a post hoc analysis confirmed that the study’s statistical power was limited, being sensitive primarily to large genetic effects. Most of the nominally significant associations were underpowered. The observed overfitting (training R^2^ ≈ 1.0 vs. test R^2^ ≈ 0.54) underscores that the identified biomarker associations (IL-8, IL-15, albumin) are preliminary hypotheses rather than validated predictors, and the cluster-then-predict approach requires validation in larger, independent cohorts. Furthermore, the lack of data on hormonal status in women, recent weight history, and medication use represents a limitation, as these factors are known to influence systemic inflammation and could act as unmeasured confounders. The cross-sectional design of the study does not allow for the establishment of causal relationships, revealing only statistical associations. All findings should be considered hypothesis-generating pending validation in larger cohorts.

## 5. Conclusions

In the present study, a search for associations between polymorphic variants of eight genes and the cytokine profile in obesity was conducted. A number of nominally significant associations were identified, which can be considered as a basis for distinguishing potential genetic markers associated with different patterns of systemic inflammation.

The analysis allowed for a conditional grouping of the studied polymorphisms according to the nature of the identified associations. *COL1A1* (rs1107946) and *MMP9* (rs17576) were classified as potentially favorable markers, associated with a decrease in pro-inflammatory or an increase in anti-inflammatory potential. For *COL1A1*, the heterozygous CA genotype was associated with decreased IL-6 levels, and for *MMP9*, the AG genotype was associated with increased IL-10 levels.

The opposite spectrum of associations, indicating a link with an enhanced pro-inflammatory background, was found for the polymorphisms *AGTR1* (rs5186) and *MTHFR* (rs1801131). The AC genotype of the *AGTR1* gene was associated with increased levels of TNF-α and IP-10 (CXCL10), while for *MTHFR*, the AC genotype was associated with increased levels of the chemokine MIP-1β (CCL4). These associations are consistent with the known pro-inflammatory role of the corresponding biological pathways.

A more complex, pleiotropic pattern of associations was demonstrated by the polymorphisms *MMP2* (rs243865) and *NOS3* (rs1799983). For *MMP2* (CT genotype), an association with increased levels of IL-7 and Fractalkine (CX3CL1) was identified, and for *NOS3* (GT genotype), an association with increased levels of the pro-inflammatory chemokines MCP-1 and Eotaxin was found, which may reflect its involvement in vascular inflammation.

Thus, the study describes a complex of associations between polymorphisms in genes of various functional profiles and markers of inflammation in obesity. The obtained data, which are hypothesis-generating in nature, form a basis for further investigation of the role of the identified genetic variants as markers for stratifying patients with obesity according to the risk of developing inflammatory complications.

Our analysis provides evidence against the homogeneous view of obesity by identifying distinct patient subpopulations through cluster analysis. In one well-defined subgroup, we established an exploratory predictive model (test R^2^ = 0.537) for BMI using specific biomarkers—IL-8, IL-15 and albumin—suggesting a distinct inflammatory signature. Meanwhile, the complex, non-linear relationships in the other subgroup highlight the necessity of such stratification for accurate biological interpretation and future research.

These findings underscore the complex nature of the genetic regulation of the inflammatory response in obesity and suggest potential markers for the stratification of complication risks.

## Data Availability

The data used to support the findings of this study are available from the corresponding author upon request.

## Supplementary Materials

The following supporting information can be downloaded at: https://www.mdpi.com/article/10.3390/biomedicines14020447/s1, Figure S1. Scatter plots illustrating significant associations between cytokine levels and body mass index (BMI). (a) Positive correlation between IL-17E/IL-25 and BMI (Spearman’s r = 0.44, FDR-adjusted *p* = 0.023). (b) Negative correlation between IL-27 and BMI (Spearman’s r = −0.40, FDR-adjusted *p* = 0.023). Trend lines represent the linear regression fit; Figure S2. Spearman correlation matrix of demographic, anthropometric and biochemical parameters; Figure S3. Pearson correlation matrix of demographic, anthropometric and biochemical parameters; Figure S4. Boxplot of standardized features with outliers; Figure S5. Feature correlations after preprocessing; Figure S6. Feature selection using Variance Inflation Factor (VIF); Figure S7. Model comparison for Cluster 1; Figure S8. Learning curves for Catboost and Ridge models; Table S1. LOD Values (Overnight Protocol, MinDC + 2SD, pg/mL); Table S2. Baseline Characteristics of the Study Cohort Stratified by BMI Category; Table S3. Robustness analysis of SNP-cytokine associations; Table S4. Associations of cytokine levels with BMI and age; Table S5. Genotype distribution and Hardy–Weinberg equilibrium analysis; Table S6. Results of predictive modelling for Cluster 1.

## Abbreviations

The following abbreviations are used in this manuscript:

BMI	Body Mass Index
SNP	Single-Nucleotide Polymorphism
MMP	Matrix Metalloproteinase
ECM	Extracellular Matrix
HWE	Hardy–Weinberg Equilibrium
FDR	False Discovery Rate
GLM	Generalized Linear Model
OLS	Ordinary Least Squares
VIF	Variance Inflation Factor
IQR	Interquartile Range
KNN	k-Nearest Neighbors
MAE	Mean Absolute Error
SHAP	SHapley Additive exPlanations
