# Supplementary material for: Elucidating Genetic Drivers of Chronic Inflammation in Obesity
Source: Biomedicines. 2026 Feb 17;14(2):447. doi: 10.3390/biomedicines14020447 (PMC12938195; doi:10.3390/biomedicines14020447)
Supplement: Supplementary file 1 [file biomedicines-14-00447-s001.zip › biomedicines-4126936-supplementary.pdf]

Supplementary Materials

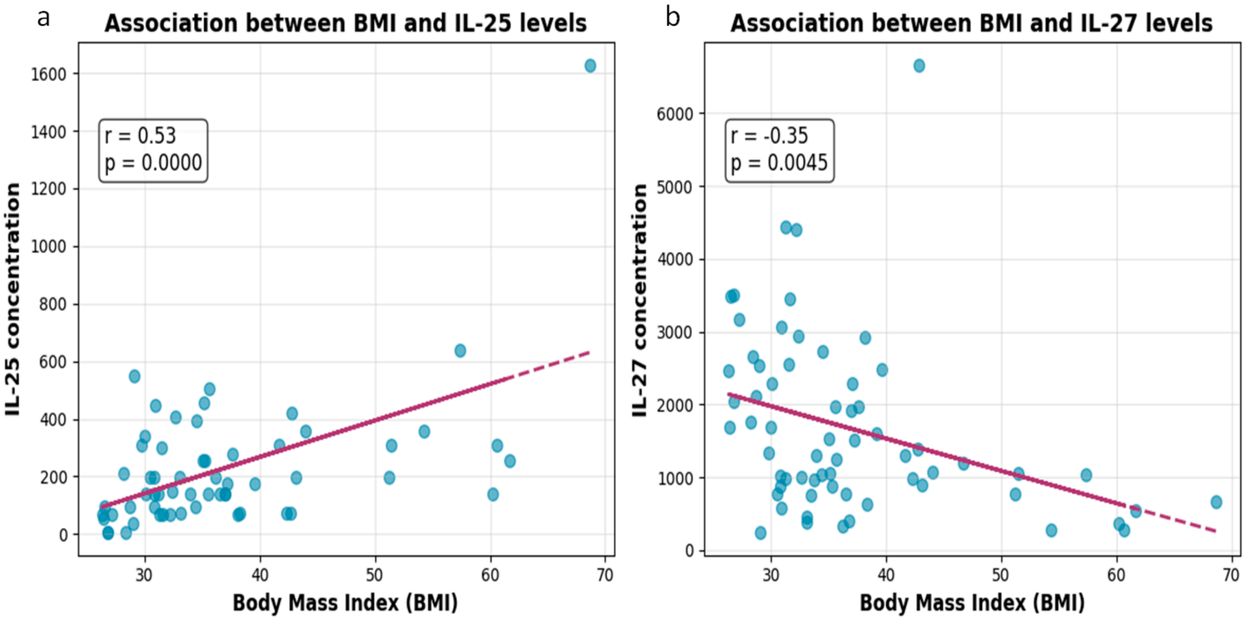

Figure S1. Scatter plots illustrating significant associations between cytokine levels and body mass index (BMI). (a) Positive correlation between IL-17E/IL-25 and BMI (Spearman's r = 0.44, FDR-adjusted p = 0.023). (b) Negative correlation between IL-27 and BMI (Spearman's r = -0.40, FDR-adjusted p = 0.023). Trend lines represent the linear regression fit.

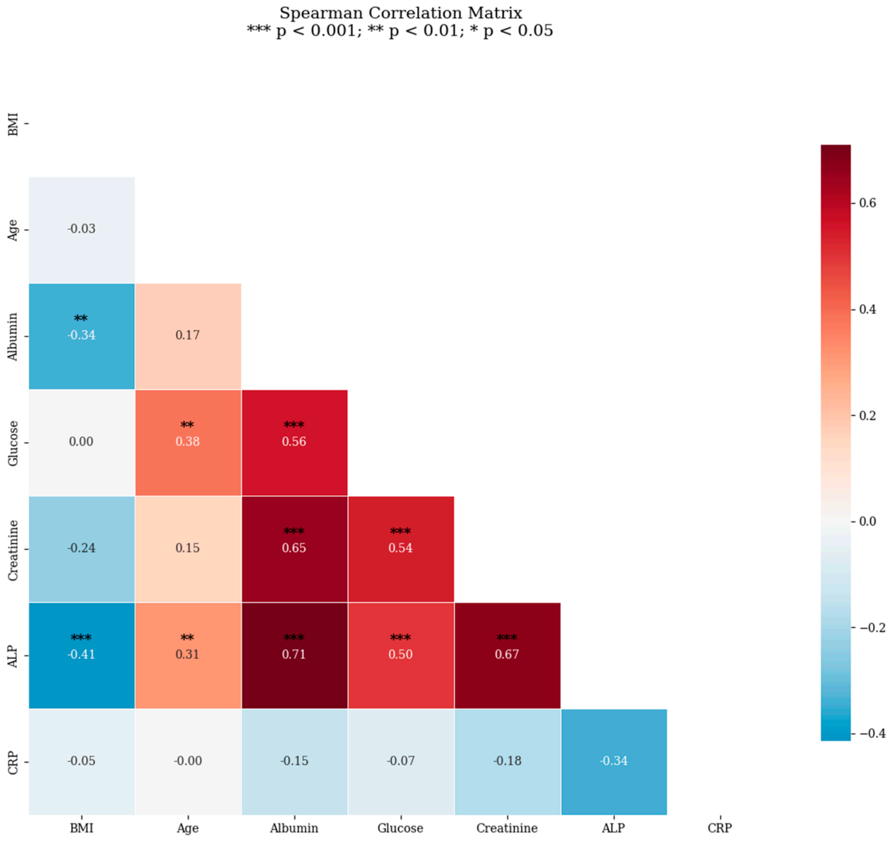

Figure S2. Spearman correlation matrix of demographic, anthropometric and biochemical parameters.

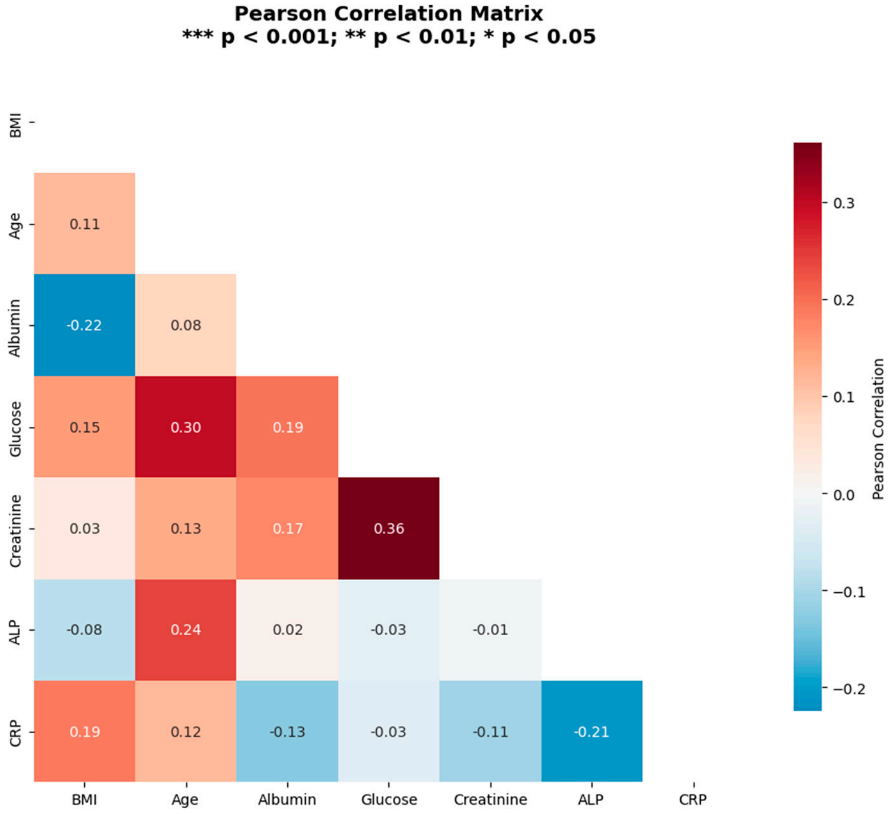

Figure S3. Pearson correlation matrix of demographic, anthropometric and biochemical parameters.

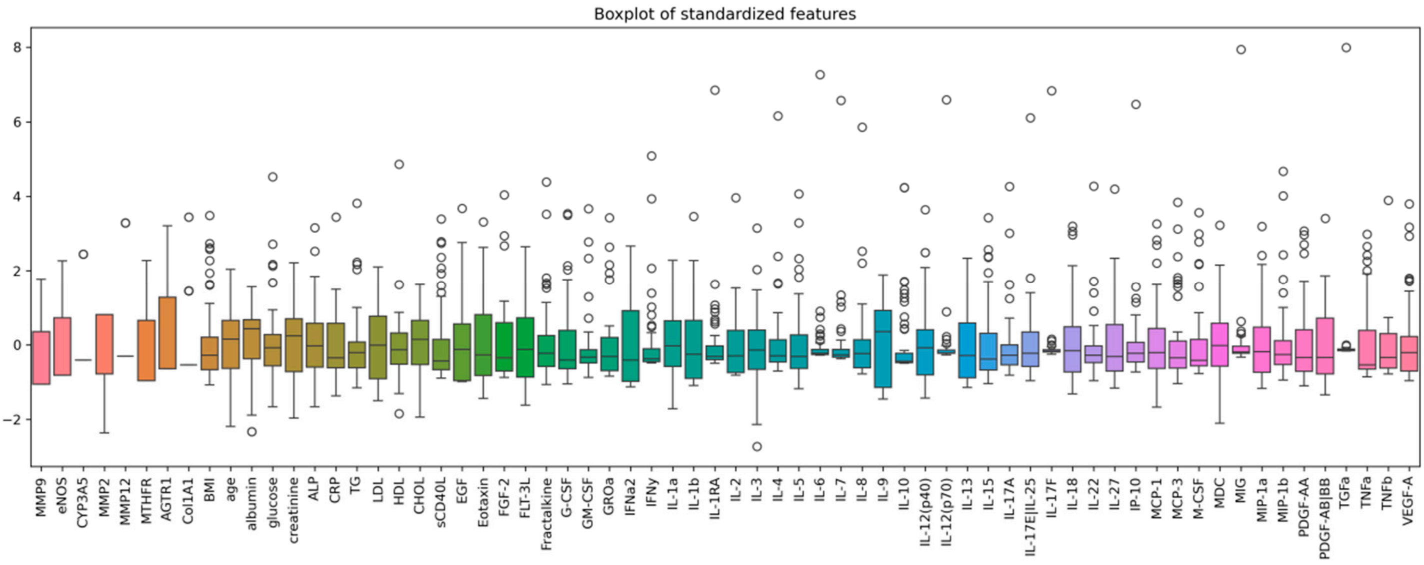

Figure S4. Boxplot of standardized features with outliers.

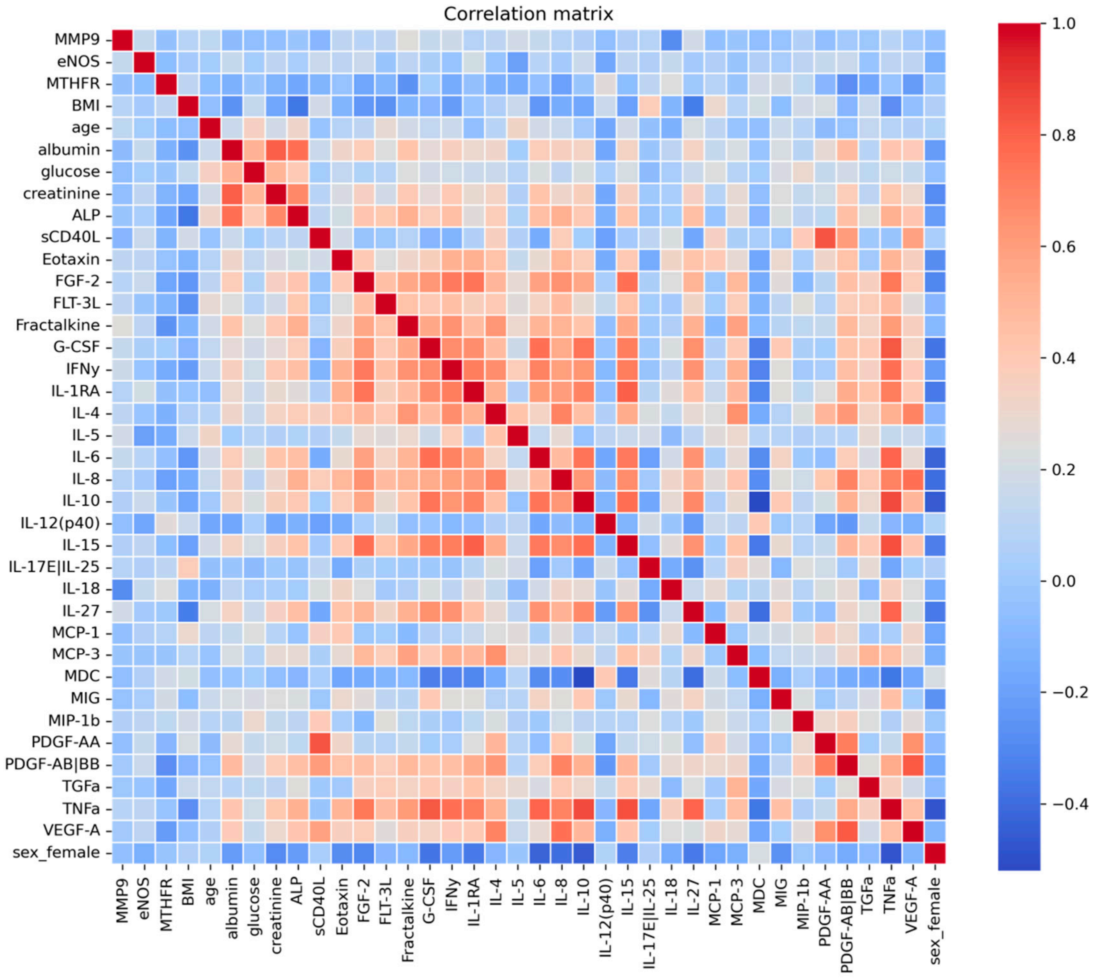

Figure S5. Feature correlations after preprocessing.

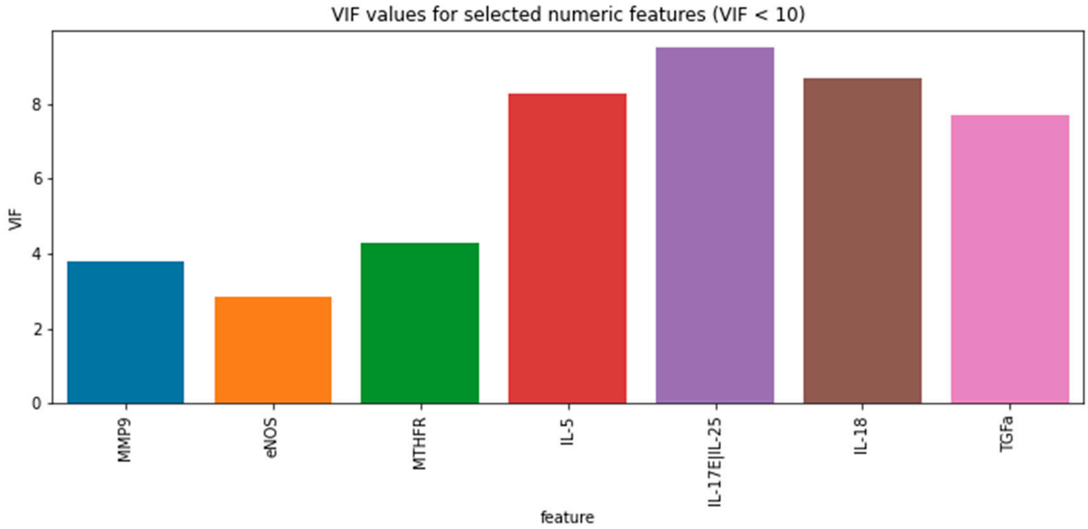

Figure S6. Feature selection using Variance Inflation Factor (VIF).

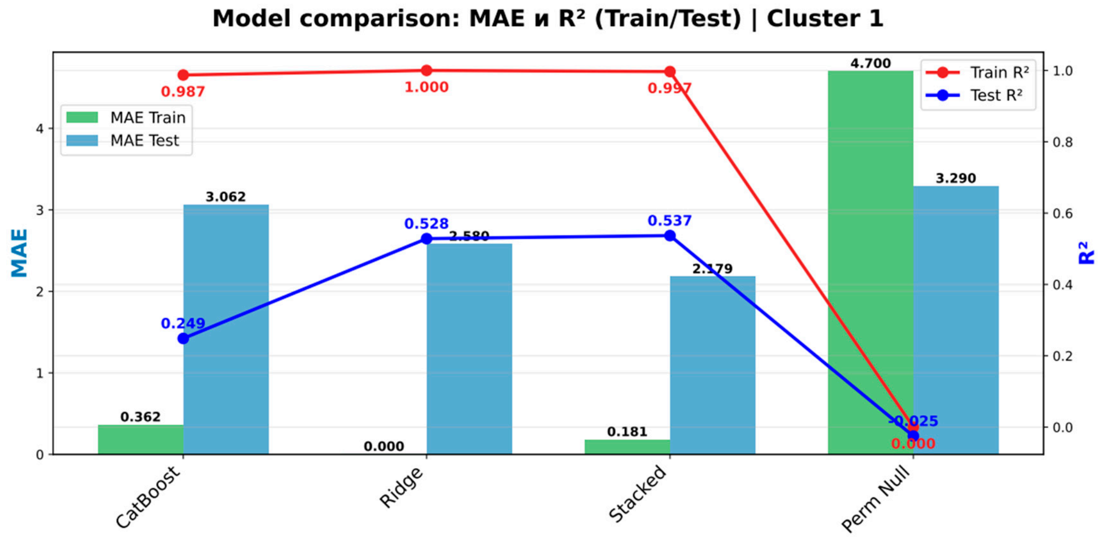

Figure S7. Model comparison for Cluster 1.

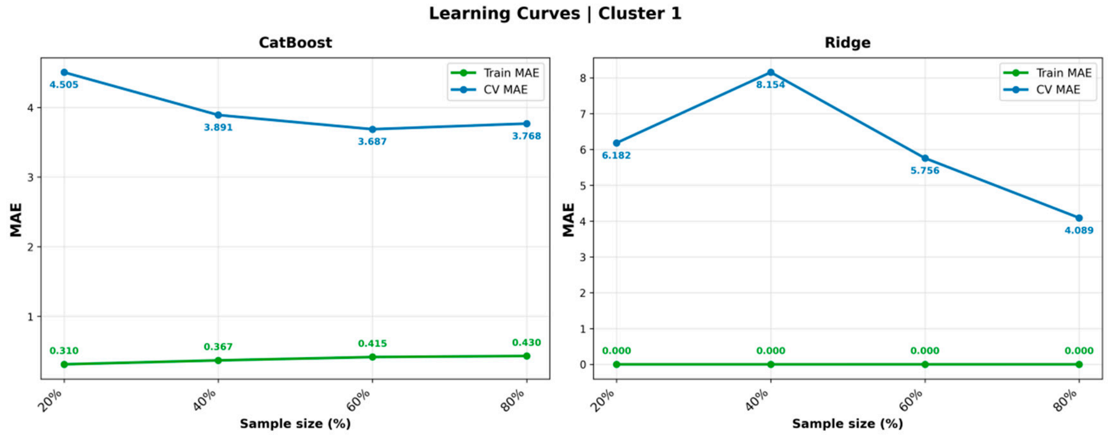

Figure S8. Learning curves for Catboost and Ridge models.

Table S1. LOD Values (Overnight Protocol, MinDC+2SD, pg/mL)

Analyte	LOD	Analyte	LOD	Analyte	LOD
**sCD40L**	5.95	IL-4	0.28	IL-17A	1.16
**EGF**	3.42	IL-5	0.21	IL-17E/IL-25	52.07
**Eotaxin**	3.26	IL-6	0.20	IL-17F	31.34
**FGF-2**	27.77	IL-7	0.20	IP-10	1.78
**FLT-3L**	0.80	IL-8	0.58	VEGF-A	3.24
**Fractalkine**	34.41	IL-9	0.39	VEGF-B	10.05
**G-CSF**	5.37	IL-10	3.48	VEGF-C	35.82
**GM-CSF**	2.05	IL-12 (p40)	6.31	VEGF-D	6.69
**GROα**	1.42	IL-12 (p70)	1.20	VEGF-E	6.35
**IFNα2**	9.49	IL-13	3.92	VEGF-F	4.64
**IFNγ**	1.42	IL-15	1.08	VEGF-G	6.84
**IL-1β**	0.79	IL-1RA	1.53	VEGF-H	12.94
**IL-2**	0.46	IL-10*	0.68	VEGF-I	42.76
**IL-3**	0.39	IL-12 (p40)*	13.88	VEGF-J	2.66

Table S2. Baseline Characteristics of the Study Cohort Stratified by BMI Category

Characteristic	Overweight (25-29.9)	Obesity I (30-34.9)	Obesity II (35-39.9)	Obesity III (≥40)	Overall
N	17	22	17	15	71
Female	10 (58.8%)	19 (86.4%)	15 (88.2%)	11 (73.3%)	55 (77.5%)
Male	7 (41.2%)	3 (13.6%)	2 (11.8%)	4 (26.7%)	16 (22.5%)
Age, years	52.2 ± 15.7	50.2 ± 13.8	49.7 ± 13.1	51.4 ± 15.5	50.8 ± 14.2
Age range	21-80	24-71	24-67	20-71	20-80
BMI, kg/m^2	27.9 ± 1.1	32.0 ± 1.4	36.9 ± 1.4	51.2 ± 8.8	36.3 ± 9.4
Current smokers	2 (11.8%)	4 (18.2%)	1 (5.9%)	4 (26.7%)	11 (15.5%)
Alcohol users	3 (17.6%)	6 (27.3%)	3 (17.6%)	1 (6.7%)	13 (18.3%)
Impaired fasting glucose (5.6-6.9 mmol/L)	3 (17.6%)	5 (22.7%)	2 (11.8%)	2 (13.3%)	12 (16.9%)
Hyperglycemia (≥7.0 mmol/L)	0 (0.0%)	1 (4.5%)	1 (5.9%)	4 (26.7%)	6 (8.5%)
TG >1.7 mmol/L	1 (5.9%)	1 (4.5%)	4 (23.5%)	2 (13.3%)	8 (11.3%)
LDL >3.0 mmol/L	2 (11.8%)	3 (13.6%)	5 (29.4%)	6 (40.0%)	16 (22.5%)
HDL <1.0 mmol/L	0 (0.0%)	3 (13.6%)	3 (17.6%)	2 (13.3%)	8 (11.3%)
Total cholesterol >5.2 mmol/L	1 (5.9%)	2 (9.1%)	4 (23.5%)	4 (26.7%)	11 (15.5%)
Dyslipidemia (any abnormality)	3 (17.6%)	7 (31.8%)	10 (58.8%)	8 (53.3%)	28 (39.4%)

CRP >5 mg/L	5 (29.4%)	4 (18.2%)	0 (0.0%)	1 (6.7%)	10 (14.1%)
Type 1 diabetes	0 (0.0%)	1 (4.5%)	0 (0.0%)	0 (0.0%)	1 (1.4%)
Type 2 diabetes	0 (0.0%)	0 (0.0%)	0 (0.0%)	2 (13.3%)	2 (2.8%)
Arterial hypertension	3 (17.6%)	5 (22.7%)	3 (17.6%)	8 (53.3%)	19 (26.8%)
Cardiovascular disease	0 (0.0%)	2 (9.1%)	1 (5.9%)	2 (13.3%)	5 (7.0%)
Thyroid disorders	1 (5.9%)	3 (13.6%)	1 (5.9%)	1 (6.7%)	6 (8.5%)
Pancreatitis	1 (5.9%)	2 (9.1%)	3 (17.6%)	0 (0.0%)	6 (8.5%)
Gastritis	1 (5.9%)	5 (22.7%)	2 (11.8%)	0 (0.0%)	8 (11.3%)
Cholecystitis	0 (0.0%)	5 (22.7%)	1 (5.9%)	0 (0.0%)	6 (8.5%)
Liver disease	0 (0.0%)	0 (0.0%)	0 (0.0%)	1 (6.7%)	1 (1.4%)
Respiratory disease	2 (11.8%)	0 (0.0%)	2 (11.8%)	1 (6.7%)	5 (7.0%)
Musculoskeletal disorders	1 (5.9%)	5 (22.7%)	5 (29.4%)	3 (20.0%)	14 (19.7%)
Allergic diseases	0 (0.0%)	0 (0.0%)	1 (5.9%)	0 (0.0%)	1 (1.4%)

Table S3. Robustness analysis of SNP-cytokine associations

SNP	Cytokine	Model	β (95% CI)	p_OLS	p_BoxCox	p_Rank	p_Huber	All_sig
COL1A1	IL-6	CA vs CC	-1.335 [-2.224, -0.446]	0,0038	0.0031	0.0040	0.0092	Yes
MTHFR	MIP-1b	AC vs AA	0.363 [0.113, 0.612]	0,005	0.0036	0.0061	0.0026	Yes
NOS3	Eotaxin	GT vs GG	0.792 [0.232, 1.351]	0,0063	0.0066	0.0047	0.0093	Yes
NOS3	MCP-1	GT vs GG	0.552 [0.160, 0.945]	0,0066	0.0075	0.0030	0.0179	Yes
AGTR1	IP-10	AC vs AA	0.992 [0.261, 1.723]	0,0092	0.0001	0.0001	0.0002	Yes
MMP2	IL-7	CT vs TT	0.863 [0.167, 1.559]	0,0165	0.0173	0.0247	0.0115	Yes
MMP2	Fractalkine	CT vs TT	0.559 [0.103, 1.016]	0,0178	0.0169	0.0062	0.0046	Yes
MMP9	IL-10	AA vs AG	-0.848 [-1.602, -0.094]	0,0281	NA	0.0468	0.0498	Yes*
AGTR1	TNFa	AC vs AA	0.556 [0.033, 1.079]	0,038	0.0349	0.0411	0.0723	No

		Add_AIC	Dom_AIC	Rec_AIC	Best_model	ΔAIC	Balanced	Robust_conf
COL1A1	IL-6	283,5	281,5	285,5	Dom	2	Yes	Yes
MTHFR	MIP-1b	224,2	222,2	226,2	Dom	2	Yes	Yes
NOS3	Eotaxin	234	232	236	Dom	2	Yes	Yes
NOS3	MCP-1	279,4	277,4	281,4	Dom	2	Yes	Yes
AGTR1	IP-10	136,5	134,5	138,5	Dom	2	Yes	Yes
MMP2	IL-7	132,6	134,6	130,6	Rec	4	Yes	Yes
MMP2	Fractalki	99,9	101,9	97,9	Rec	4	Yes	Yes

	ne							
MMP9	IL-10	277,6	275,6	279,6	Dom	2	Yes	Yes
AGTR1	TNFa	113,7	111,7	115,7	Dom	2	Yes	Yes

Table S4. Associations of cytokine levels with BMI and age

Cytokine	BMI_Correlation	BMI_P_Value	Age_Correlation	Age_P_Value	BMI_FDR_P_Value	BMI_FDR_Significant	Age_FDR_P_Value	Age_FDR_Significant
sCD40L	0,309486	0,012828	-0,17284	0,165186	0,150725	no	0,408653	no
EGF	0,07843	0,604388	-0,01274	0,931536	0,728365	no	0,951787	no
Eotaxin	-0,15	0,236776	0,137735	0,270091	0,483846	no	0,505174	no
FGF-2	-0,23977	0,077871	0,277621	0,036541	0,261425	no	0,250629	no
FLT-3L	-0,29321	0,01969	0,329245	0,007405	0,18509	no	0,250629	no
Fractalkine	-0,1534	0,230028	0,260205	0,036319	0,483846	no	0,250629	no
G-CSF	-0,19395	0,127738	0,203615	0,103772	0,375229	no	0,336066	no
GM-CSF	0,080538	0,655929	0,173423	0,319106	0,751919	no	0,54375	no
GROa	-0,28676	0,076735	-0,01448	0,93027	0,261425	no	0,951787	no
IFNa2	-0,0769	0,591691	0,227097	0,101983	0,728365	no	0,336066	no
IFNy	-0,27694	0,040669	0,257217	0,053414	0,245414	no	0,313805	no
IL-1a	0,149598	0,326678	0,207943	0,160742	0,562301	no	0,408653	no
IL-1b	-0,15217	0,301835	0,112911	0,434974	0,562301	no	0,601287	no
IL-1RA	-0,02834	0,834226	0,058507	0,659817	0,884322	no	0,783087	no
IL-2	-0,17646	0,388511	0,262852	0,185295	0,589032	no	0,435444	no
IL-3	0,282951	0,069417	0,246149	0,107255	0,261425	no	0,336066	no
IL-4	0,10465	0,438507	0,082428	0,534828	0,644057	no	0,661498	no
IL-5	0,109164	0,386697	0,29595	0,015032	0,589032	no	0,250629	no
IL-6	-0,17834	0,192679	0,276513	0,037328	0,466476	no	0,250629	no
IL-7	-0,26502	0,065718	0,161989	0,256092	0,261425	no	0,505174	no
IL-8	-0,12377	0,346113	0,179639	0,162387	0,562301	no	0,408653	no
IL-9	0,181168	0,346952	0,330026	0,074896	0,562301	no	0,32001	no
IL-10	-0,17718	0,183352	-0,00184	0,988891	0,466476	no	0,988891	no

IL-12(p40)	0,121352	0,343424	-0,08857	0,482933	0,562301	no	0,630496	no
IL-12(p70)	-0,11334	0,458503	0,164779	0,268364	0,65265	no	0,505174	no
IL-13	0,035583	0,846691	0,14803	0,411006	0,884322	no	0,601287	no
IL-15	-0,25728	0,047204	0,215431	0,092647	0,245414	no	0,336066	no
IL-17A	0,21043	0,1985	-0,07029	0,666457	0,466476	no	0,783087	no
IL-17E|IL-25	0,43929	0,0005	-0,11356	0,383545	0,023195	да	0,586238	no
IL-17F	0,172662	0,251189	0,181041	0,21816	0,491912	no	0,488264	no
IL-18	-0,08866	0,486016	-0,10831	0,386667	0,65265	no	0,586238	no
IL-22	0,286686	0,147111	0,264727	0,1652	0,406718	no	0,408653	no
IL-27	-0,40217	0,000987	0,26427	0,032017	0,023195	да	0,250629	no
IP-10	0,037508	0,787721	0,147056	0,279458	0,860998	no	0,505174	no
MCP-1	0,243799	0,052216	0,090584	0,469476	0,245414	no	0,630439	no
MCP-3	-0,01952	0,881282	0,012162	0,92463	0,90044	no	0,951787	no
M-CSF	-0,22374	0,10729	0,135504	0,323936	0,336176	no	0,54375	no
MDC	0,16014	0,20994	-0,03985	0,75262	0,469866	no	0,847226	no
MIG	-0,08091	0,525072	0,279539	0,023019	0,666984	no	0,250629	no
MIP-1a	-0,06915	0,636861	0,25688	0,068807	0,748312	no	0,32001	no
MIP-1b	0,252454	0,04592	0,039108	0,757096	0,245414	no	0,847226	no
PDGF-AA	0,257237	0,040169	-0,14936	0,231311	0,245414	no	0,494165	no
PDGF-AB|BB	-0,04322	0,734508	-0,01332	0,915449	0,82195	no	0,951787	no
TGFa	0,090346	0,481321	0,111962	0,374568	0,65265	no	0,586238	no
TNFa	-0,31447	0,011383	0,224746	0,069639	0,150725	no	0,32001	no
TNFb	-0,1533	0,507064	0,149123	0,518833	0,662001	no	0,659059	no
VEGF-A	-0,00971	0,939317	0,100086	0,423957	0,939317	no	0,601287	no

Table S5. Genotype distribution and Hardy-Weinberg equilibrium analysis

	Group	n	Genotypes	HWE p-value	Between-group p-value
MMP9	Obesity	72	AG: 31 (43.1%); AA: 30 (41.7%); GG: 11 (15.3%)	0.528	0.185
	Control	52	AG: 31 (59.62%); AA: 16 (30.77%); GG: 5 (9.6%)	0.073	
eNOS	Obesity	71	GG: 38 (53.5%); GT: 27 (38.0%); TT: 6 (8.5%)	0.701	0.173
	Control	51	GT: 22 (43.1%); GG: 20 (39.2%); TT: 9 (17.6%)	0.497	
CYP3A5	Obesity	43	AA: 36 (83.7%); AG: 7 (16.3%)	0.561	0.609
	Control	55	AA: 43 (78.2%); AG: 12 (21.8%)	0.364	
MMP2	Obesity	43	TT: 24 (55.8%); CT: 15 (34.9%); CC: 4 (9.3%)	0.472	0.812
	Control	21	TT: 12 (57.1%); CT: 8 (38.1%); CC: 1 (4.8%)	0.819	
MMP12	Obesity	72	AA: 66 (91.7%); AG: 6 (8.3%)	0.712	1.000
	Control	49	AA: 45 (91.8%); AG: 4 (8.2%)	0.766	
MTHFR	Obesity	72	AC: 34 (47.2%); AA: 33 (45.8%); CC: 5 (6.9%)	0.339	0.363
	Control	52	AA: 28 (53.8%); AC: 23 (44.2%); CC: 1 (1.9%)	0.128	
AGTR1	Obesity	43	AA: 30 (69.8%); AC: 12 (27.9%); CC: 1 (2.3%)	0.876	0.673
	Control	21	AA: 16 (76.2%); AC: 4 (19.0%); CC: 1 (4.8%)	0.309	
Col1A1	Obesity	72	CC: 54 (75.0%); CA: 16 (22.2%); AA: 2 (2.8%)	0.547	0.771
	Control	52	CC: 36 (69.2%); CA: 14 (26.9%); AA: 2 (3.8%)	0.668	

Table S6. Results of predictive modelling for Cluster 1.

Model	MAE_train	R^2_train	MAE_test	R^2_test
CatBoost	0.362	0.987	3.062	0.249
Ridge	0.000	1.000	2.580	0.528
Stacked	0.181	0.997	2.179	0.537
Perm Null	4.700	0.000	3.290	-0.025
